# Recent Advances in Hydrogel Technology in Delivering Mesenchymal Stem Cell for Osteoarthritis Therapy

**DOI:** 10.3390/biom14070858

**Published:** 2024-07-17

**Authors:** Xiangjiang Wang, Wentao He, Hao Huang, Jiali Han, Ruren Wang, Hongyi Li, Ying Long, Guiqing Wang, Xianjing Han

**Affiliations:** 1 The Affiliated Qingyuan Hospital (Qingyuan People’s Hospital), Guangzhou Medical University, Qingyuan 511518, China; xiangjiang724@gzhmu.edu.cn (X.W.); 2023210862@stu.gzhmu.edu.cn (W.H.); 2022210885@stu.gzhmu.edu.cn (J.H.); 2023210859@stu.gzhmu.edu.cn (R.W.); lhy13553958184@gmail.com (H.L.); ly15975884327@gmail.com (Y.L.); 2 Key Laboratory of Optoelectronic Devices and Systems of Ministry of Education and Guangdong Province, Collage of Physics and Optoelectronics Engineering, Shenzhen University, Shenzhen 518060, China; dchty588@gmail.com

**Keywords:** mesenchymal stem cell, hydrogel, osteoarthritis, articular cartilage degradation

## Abstract

Osteoarthritis (OA), a chronic joint disease affecting over 500 million individuals globally, is characterized by the destruction of articular cartilage and joint inflammation. Conventional treatments are insufficient for repairing damaged joint tissue, necessitating novel therapeutic approaches. Mesenchymal stem cells (MSCs), with their potential for differentiation and self-renewal, hold great promise as a treatment for OA. However, challenges such as MSC viability and apoptosis in the ischemic joint environment hinder their therapeutic effectiveness. Hydrogels with biocompatibility and degradability offer a three-dimensional scaffold that support cell viability and differentiation, making them ideal for MSC delivery in OA treatment. This review discusses the pathological features of OA, the properties of MSCs, the challenges associated with MSC therapy, and methods for hydrogel preparation and functionalization. Furthermore, it highlights the advantages of hydrogel-based MSC delivery systems while providing insights into future research directions and the clinical potential of this approach.

## 1. Introduction

Osteoarthritis (OA) is a prevalent chronic joint disease characterized by damaged articular cartilage tissue and the onset of joint inflammation, resulting in pain, functional limitations, and diminished quality of life for patients [1,2,3]. Conventional treatment methods are inadequate in repairing damaged joint cartilage tissue, highlighting the need for effective therapeutic approaches that facilitate joint tissue regeneration.

Mesenchymal stem cell (MSC) regenerative therapies have emerged as promising strategies for repairing damaged tissues and organs. MSCs can self-renew and differentiate into a variety of cell types, including chondrocytes and osteoblasts [4,5]. Moreover, their wide availability from diverse sources, ease of accessibility, and expandability in vitro make them a focal point in OA treatment research. However, MSC therapy for OA encounters significant challenges. For instance, the reduced viability and increased susceptibility to apoptosis of MSCs within the ischemic and hypoxic environment of the joint cavity hinder their therapeutic efficacy [6]. Additionally, the shear stress generated by the injection needle during MSC delivery into the joint cavity can compromise their viability [7]. Furthermore, individually injected MSCs may not fully differentiate into the desired cell types [8]. Henceforth, there is an urgent need for delivery techniques that ensure an optimal differentiation environment within the joint cavity while maintaining MSC viability during administration.

Hydrogels, as versatile carriers for drugs and cells, offer a three-dimensional scaffold structure that mimics the physiological environment of tissues. They have been demonstrated to be able to support cell viability and differentiation in various disease studies [9]. Furthermore, hydrogels exhibit excellent biocompatibility and biodegradability, making them an ideal choice for delivering MSCs in the treatment of osteoarthritis [10]. 

Here, we comprehensively reviewed the pathological features of OA, the properties of MSCs, and their applications in OA treatment. We also highlight the challenges associated with MSC therapy for OA. Subsequently, we delve into hydrogel preparation, functionalization methods, and their diverse biomedical applications. Finally, we discuss the advantages of hydrogels in delivering MSCs and review reported research cases involving applying hydrogels for MSC delivery in the treatment of OA. Additionally, we offer insights into future research directions and the promising clinical prospects of this approach.

## 2. Osteoarthritis (OA)

### 2.1. Pidemiological Characteristics and Physiopathological Mechanisms of OA

OA, a degeneration of the joints, primarily affects individuals aged 50 and older, with a higher incidence among females compared to males [11,12]. It is estimated that the global population of OA patients reaches as high as 500 million. By 2032, the prevalence of OA is projected to increase by 26.6% to 29.5% [13]. OA not only affects the patients’ health physically and mentally but also causes economic loss for both individuals and society. The average annual treatment costs per OA patient range from USD 700 to USD 15,600 [14].

**Figure 1:**
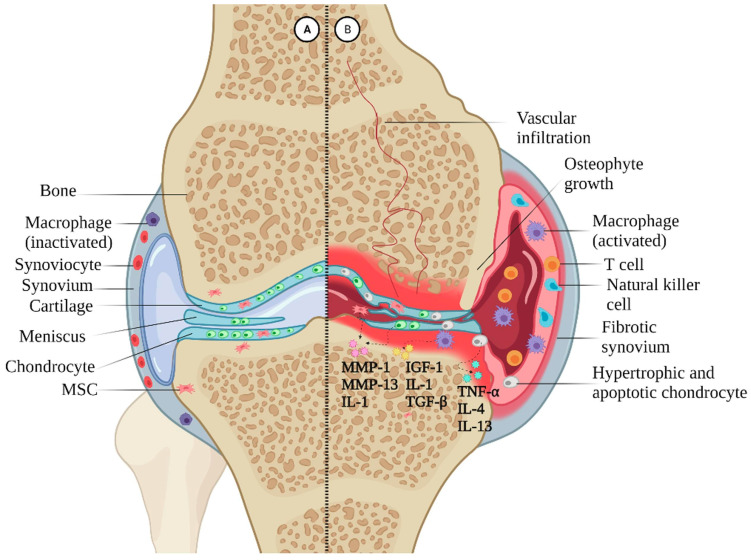
Schematic illustration of the physiopathology of OA. Part A indicates the healthy joint while Part B indicates the osteoarthritic joint. Reprinted with permission from Ref. [22]. Copyright 2022 Elsevier.

OA is a chronic degenerative disease that impacts the whole joint, including bones, articular tissue, the synovium, subchondral bone, the meniscus, and ligaments. The primary characteristics of osteoarthritis involve the degeneration and deterioration of joint cartilage, the meniscus, and ligaments, along with inflammation and sclerosis of synovial tissues and the presence of subchondral bone cysts [15]. Traditionally viewed as a passive degenerative joint disease or a consequence of long-term mechanical wear and tear, emerging perspectives indicate that the OA process is active and dynamic. It is primarily driven by an imbalance between joint degradation and repair [16]. Initially, erosion starts on the cartilage surface and gradually penetrates into the calcified articular cartilage zone [17]. The avascular nature of cartilage tissue, coupled with limited chondrocyte proliferative capacity and impaired intrinsic repair mechanisms, exacerbates the deterioration of the cartilage layer [18,19]. Concurrently, the physical stress resulting from cartilage defects triggers pathological subchondral bone remodeling, further compromising the integrity of the cartilage layer. Ultimately within the cartilage microenvironment it contributes to osteophyte formation around the joint periphery [15]. (Figure 1).

Studies have demonstrated that the progression of OA is influenced by inflammatory factors, metalloproteinases, cellular senescence, estrogen, and biomechanical imbalances within the joint cartilage. These factors collectively contribute to local cartilage damage, osteophyte formation, subchondral bone remodeling, and excessive synovial proliferation. These pathological changes profoundly impact the patients’ quality of life and serve as crucial indicators of OA progression [20,21].

### 2.2. Current Dilemma in OA Treatment

Currently, due to the avascular nature of articular cartilage tissues and the low self-renewing capacity of chondrocytes, structural damage to articular cartilage cannot self-heal. This is considered a fundamental factor in the progression and difficult-to-treat nature of OA [23]. There are no effective methods or drugs for the comprehensive treatment of OA. At present, treatment approaches for OA can be categorized into physical therapy, pharmacotherapy, and surgical intervention, as summarized in Table 1. However, the majority of current clinical drugs and treatment modalities only provide symptomatic relief by reducing joint inflammation and pain, thereby slowing down the progression of OA. They are unable to achieve complete healing of the damaged cartilage or cure OA [24].

Moreover, only a small number of patients achieve partial regeneration of articular cartilage through non-surgical treatments. However, the regeneration process is time-consuming, and the newly formed cartilage tissue often lacks adequate hardness and compressive strength [25]. It is crucial to be aware of the potential serious side effects associated with drug treatments. For instance, long-term oral administration of nonsteroidal anti-inflammatory drugs (NSAIDs) may lead to the development of gastrointestinal ulcers and bleeding. Moreover, drugs administered via intra-articular injection have limited residence time within the joint cavity, leading to low drug utilization and the need for repeated administration, thereby increasing the risk of treatment-related harm [26].

Surgical intervention is often necessary for patients with moderate to advanced OA. However, surgical treatments are invasive and may carry potential risks, such as infection and thrombosis, posing significant harm to the overall well-being of patients [27,28]. Therefore, there is an urgent need to develop new therapeutic drugs or approaches that can achieve cartilage repair and regeneration and ultimately cure OA.

**Table 1:** Common treatments for osteoarthritis.

Treaments		Therapeutic Effects	Ref.
Pharmacotherapy	Diacerein	Induces chondrogenesis; has analgesic, anti-inflammatory and antipyretic effects; and improves joint function in patients with osteoarthritis	[29,30,31,32]
	Chondroitin/glucosamine	Pain reliever, promotes cartilage regeneration	
	Acetaminophen	Pain reliever	
	Opioids	Pain reliever	
	NSAIDs	Suppresses the degradation of cartilage ECM, increases ECM anabolism, and reduces chondrocytes apoptosis	
Physical modalities	Exercise, Tai Chi	Reduces weight load and maintains body balance	[33,34,35,36,37,38,39,40]
	Crutches	Reduces joint loads	
	Acupuncture, balneotherapy/spa, hydrotherapy, therapeutic ultrasound	Reduces local inflammatory stimuli by decreasing the expression of inflammatory factors, enhances the muscle strength around the knee to balance the stress	
	NMES, TENS	Relieves pain, improves blood circulation, reduces edema, promotes bone and wound healing, etc.	
Surgical treatments	Total joint arthroplasty, hemiarthroplasty, arthroscopy	Reconstruction of joints to restore normal motor function	[41,42]

## 3. Roles of MSCs in OA Therapy

### 3.1. Physiological Characteristics of MSCs

MSCs have long been of great interest in the fields of medical repairment, regeneration, and immune modulation. MSCs are non-phagocytic cells with a fibroblast-like appearance that express specific surface antigens [43,44] (Table 2). They possess potent self-renewal and multipotent differentiation capabilities, as well as remarkable immunomodulatory, anti-inflammatory, and homing properties, making them highly attractive in regenerative medicine [45,46]. The self-renewal capacity of MSCs allows them to maintain their population through cell division, while their multipotent differentiation ability enables them to differentiate into different cell types, including osteocytes, chondrocytes, and adipocytes [47,48]. Furthermore, their homing ability allows these stem cells to precisely target injured or treated areas, effectively enhancing the precision and efficiency of therapeutic interventions [49].

Initially, MSCs were primarily extracted from bone marrow. However, due to the challenges and invasiveness associated with bone marrow collection, researchers began exploring other tissue sources. Subsequently, MSCs were successfully isolated from adipose tissue, synovium, umbilical cord blood, periosteum, amniotic fluid, and membrane and perichondrium [50]. Some commonly used MSCs and their applications are summarized in Table 3. Among these, adipose-derived mesenchymal stem cells (AD-MSCs) and bone marrow-derived mesenchymal stem cells (BM-MSCs) are currently the most widely used in clinical applications. This is because both AD-MSCs and BM-MSCs are relatively easy to culture and can be obtained in large quantities [51]. They both possess immunomodulatory properties and can mediate inflammation. Additionally, they exhibit low immunogenicity, which allows for their use in mismatched or even xenogeneic environments [52,53].

Studies have found that at the single-cell level, AD-MSCs exhibit lower transcriptional heterogeneity and immunogenicity compared to BM-MSCs [54]. Additionally, they demonstrate a stronger immunosuppressive capacity and higher multipotency [47]. Furthermore, adipose-derived MSCs exhibit a faster expansion rate and higher cell survival rates, and are more likely to retain their stem cell phenotype during the culture process [55].

**Table 3:** Varieties of MSCs and their biomedical applications.

MSCs	Origin	Differentiation Potential	Applications
BM-MSCs	Bone marrow	Osteocytes, chondrocytes, and adipocytes	Nonunion fractures, spinal cord injuries, and amyotrophic lateral sclerosis (ALS) [56,57,58,59,60,61]
Placenta-MSCs	Newborn placental tissue	Osteocytes, chondrocytes, adipocytes, and smooth muscle cells	Multiple sclerosis, knee osteoarthritis, preterm infant lung disease, and ovarian function restoration [62,63,64,65,66,67]
UC-MSCs	Intervascular, perivascular, and subamniotic area of Wharton’s jelly	Osteocytes, chondrocytes, and adipocytes	Treatment of neurological disorders, cardiovascular diseases, and autoimmune diseases [68,69,70,71]
ADSCs	Adipose tissue	Osteocytes, adipocytes, chondrocytes, and smooth muscle cells	Skin regeneration, soft tissue repair, and treatment of diabetes [72,73,74,75]
Sy-MSCs	Synovial fluid in the joint cavity	Osteocytes, chondrocytes, adipocytes, muscle cells, and neurons	Osteoarthritis treatment, cartilage injuries, systemic autoimmune diseases, and tissue engineering [76,77,78,79,80,81]
DPSCs	Dental pulp tissue of permanent teeth, deciduous teeth, and wisdom teeth in adults	Osteocytes, chondrocytes, adipocytes, muscle cells, and neurons	Dental treatment, neural repair, cardiovascular diseases, and bone tissue engineering [82,83,84,85,86]
AMSCs	Amniotic membrane tissue from the placenta	Osteocytes, chondrocytes, adipocytes, and smooth muscle cells	Skeletal tissue repair, autoimmune diseases, neurodegenerative diseases, liver diseases, and corneal repair [87,88,89,90,91]

### 3.2. The Therapeutic Potential of MSCs for OA

In clinical trials, MSCs have been increasingly utilized due to their stemness and tropic functions, which provide benefits. The stemness of MSCs refers to their ability to self-renew and differentiate into tissue-specific cells, including chondrocytes, adipocytes, and osteoblasts, which can replace cells in damaged tissues. The tropic functions of MSCs refers to their ability to generate a reparative milieu via cell-to-cell contact and paracrine secretion of various bioactive factors [92,93,94]. These functions promote the immunomodulation of inflammatory cells involved in tissue regeneration (e.g., macrophages, T cells, and mast cells) and their differentiation into endogenous progenitor cells (e.g., osteoprogenitors, chondroprogenitors, etc.) [95]. Due to their versatility, MSCs have been applied in the treatment of a variety of diseases. In the context of breast cancer, MSCs have been employed in conjunction with hematopoietic stem cell transplantation during chemotherapy to facilitate rapid hematopoietic recovery in patients [96]. In a study conducted by Rojas, it was observed in a mouse model that MSCs can migrate to the lungs and differentiate into lung-like cells [97]. This mechanism allows MSCs to replace damaged cells and potentially treat lung injuries. Furthermore, Jung’s research demonstrated that transfecting MSCs with insulin-like growth factor-1 (IGF-1) can provide protection to the myocardium of rats. The transfected MSCs were found to mitigate fibrosis and apoptosis, resulting in a reduction in the size of the infarcted area [98]. These studies demonstrate the broad range of applications for MSC therapy in biomedicine.

Subsequently, MSCs have shown remarkable effectiveness in treating liver disease [99], kidney injury [100], and cartilage tissue engineering [101]. Table 4 summarizes different types of MSCs applied in OA therapy. In the following sections, this article will focus on the application and mechanisms of MSCs in OA therapy by analyzing preclinical studies and clinical trials that explore the use of AD-MSCs and BM-MSCs in treating OA.

#### 3.2.1. AD-MSCs

AD-MSCs have aroused significant interest in the treatment of osteoarthritis (OA) due to their easier accessibility compared to the traditionally used BM-MSCs. AD-MSCs offer advantages such as lower risk of donor site infection and pain, higher cell yield, and a better predictable differentiation pattern [111,112]. Additionally, studies have found that AD-MSCs exhibit histological similarities to chondroblasts in terms of extracellular matrix volume and composition, based on their chondrogenic potential. Jo et al. recruited 18 patients with knee osteoarthritis (KOA) and administered intra-articular injections of AD-MSCs for OA treatment. They found that the injection of 1.0 × 10^8^ AD-MSCs into the joint cavity improved knee joint function and alleviated pain. Furthermore, they observed cartilage regeneration and a reduction in cartilage defects through the regeneration of hyaline-like articular cartilage [113]. Spasovski et al. also found that subcutaneous AD-MSCs can improve clinical symptoms in patients with osteoarthritis. They observed a reduction in pain at 3 months, with the best outcomes achieved at 6 months [114]. Additionally, studies indicated that intra-articular (IA) injection of AD-MSCs can significantly improve joint function and alleviate pain in patients with OA, with no reported adverse events [115]. The feasibility of utilizing AD-MSCs for the treatment of OA is well supported by these successful clinical cases. Studies indicate that the therapeutic mechanism underling the application of AD-MSCs in OA involves reducing pro-inflammatory cytokines and chemokines [116], inhibiting chondrocyte apoptosis [117], suppressing hypertrophy and fibrotic phenotype transition in chondrocytes [118,119], and concurrently decreasing collagenase expression [120].

#### 3.2.2. BM-MSCs

BM-MSCs are another commonly used MSC therapy for OA. Kuroda et al. discovered that autologous transplantation of BM-MSCs promotes the repair of cartilage defects in young patients [121]. Recently, the safety of IA injection of BM-MSCs was confirmed in 12 OA patients. After a two-year follow-up, pain relief and improvement in cartilage quality were observed. In addition to promoting cartilage repair, BM-MSCs may also play a role in reducing inflammatory symptoms [122]. Zhang et al. found that co-culturing BM-MSCs with chondrocytes from patients with osteoarthritis increased the proliferation of osteoarthritic chondrocytes while suppressing their inflammatory activity [104]. Another phase I/IIa clinical trial demonstrated that autologous transplantation of BM-MSC can reduce synovial inflammation, improve knee joint function, and alleviate symptoms in patients with OA [123].

### 3.3. Difficulties of Applying MSCs in OA Treatment

The use of MSCs in OA treatment holds great promise, but several challenges must be overcome. Firstly, MSCs injected into the joint may be at high risk of starvation and death due to the avascular nature of cartilage tissue, which consequently diminishes the therapeutic efficacy of MSCs [124,125]. Additionally, injecting free MSCs without any supporting agents can be detrimental to the cells, as they are exposed to shear and stretching forces, as well as pressure changes caused by the needle, leading to a reduction in cell viability [22,126]. Furthermore, free MSCs are prone to migrate away from the injection site towards the surrounding tissues, resulting in fewer cells effectively delivering their therapeutic effects at the intended location over time [127]. Moreover, bare MSCs may not provide the ideal conditions necessary to unlock their full healing potential. Studies have shown that predifferentiated chondrogenic MSCs with transforming growth factor-b3 (TGF-b3) performed better than undifferentiated MSCs when implanted with a collagen scaffold into an ovine chronic defect model [128]. Lastly, both preclinical studies and clinical applications require a substantial number of MSCs [129]. Efficiently and rapidly expanding the MSC population is not only a critical issue for the bio-application of MSCs but also an urgent problem for other types of somatic stem cells. Therefore, there is an urgent need to optimize the delivery methods of MSCs into joints for OA therapy.

## 4. Bio-Application of Hydrogel Technologies

### 4.1. Characteristics of Hydrogel Technologies and Their Biomedical Application

**Figure 2:**
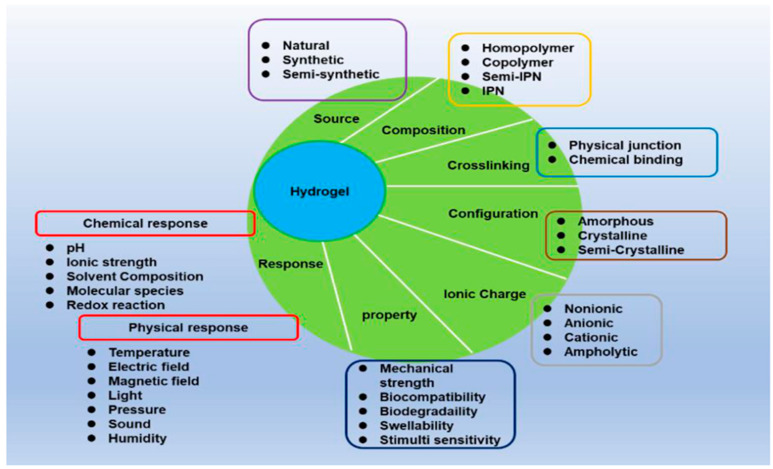
Classification of hydrogels. Reprinted with permission from Ref. [132]. Copyright 2022 MDPI.

Hydrogels, a class of unique polymer materials, are distinguished by their three-dimensional network structure formed by hydrophilic polymer chains (refer to Figure 2). These materials are known for their rapid water absorption and volumetric swelling while remaining insoluble in water [130]. The composition of hydrogels typically includes hydrophilic and hydrophobic groups, which are either chemically or physically crosslinked to create a stable network structure. This design endows hydrogels with exceptional water absorption capacity and shape-maintaining properties. Depending on the source of their monomers, hydrogels can be categorized into three types: natural hydrogels, synthetic hydrogels, and hybrid-origin hydrogels [131] (refer to Table 5).

**Figure 3:**
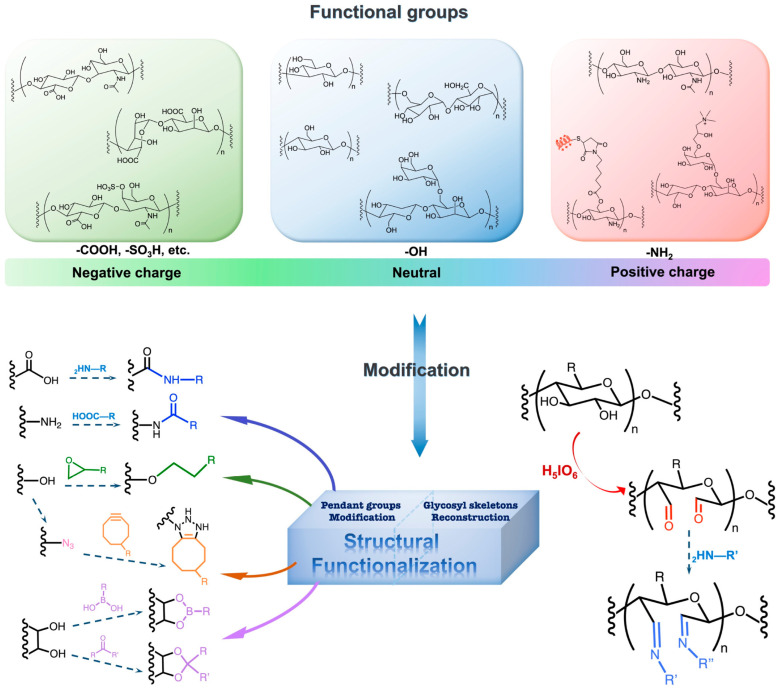
Schematic of strategies for modifying natural polysaccharide hydrogels according to their functional groups. Reprinted with permission from Ref. [147]. Copyright 2022 Elsevier.

With a specific physicochemical structure, hydrogels possess excellent properties, including biocompatibility, high water absorption, tunability, and biodegradability. Furthermore, hydrogels can be functionally modified to cater to specific application needs [146]. For example, the modification of polysaccharide-based hydrogels with polymers, including polyethylene, polyvinylpyrrolidone (PVP), and polyethylene glycol (PEG), can enhance their swelling/shrinkage responsiveness, mechanical strength, and adhesive properties (refer to Figure 3) [147]. The remarkable physicochemical attributes of hydrogels have led to their extensive application in the field of biomedicine. Currently, hydrogels are widely utilized in various applications, such as tissue engineering scaffolds, drug and cell delivery systems, controlled release systems, wound dressings, and biomedical devices. The utilization of hydrogels in these applications has been explored for various treatments, such as cancer treatment, wound healing, diabetes management, tissue regeneration, and osteoarthritis [148,149].

### 4.2. Biofabrication of Hydrogel

Hydrogels are commonly prepared using a variety of methods, each with distinct advantages, limitations, and ideal applications. These methods (refer to Table 6) include physical crosslinking, chemical crosslinking, photopolymerization, and enzymatic biocatalysis [130]. Physical crosslinking depends on non-covalent interactions between polymer chains. Techniques such as freeze-thawing create a porous structure by solidifying and recrystallizing the polymer, while ion condensation forms gels through the charge interactions of polyelectrolytes [150]. Chemical crosslinking involves the use of crosslinking agents like glutaraldehyde, diisocyanates, and natural crosslinkers such as tannic acid. These agents initiate or catalyze reactions within a polymer solution to form a crosslinked structure. This approach allows for precise control over the degree of crosslinking and the resulting physicochemical properties of hydrogels through adjusting the concentration of the crosslinking agent, the reaction time, and the temperature [151]. Photopolymerization employs photosensitive monomers and photoinitiators to initiate polymerization under ultraviolet (UV) or visible light, leading to the formation of a crosslinked network. Acrylic acid and acrylamide are commonly used as photopolymerizable monomers. The process involves dissolving these monomers and photoinitiators in a solvent, mixing them with a crosslinking agent, and exposing the prepolymer solution to light of a specific wavelength to form the hydrogel [152]. Enzymatic biocatalysis uses specific enzyme catalysts to induce crosslinking reactions within a polymer solution, a method often employed for natural polymer hydrogels such as gelatin and sodium alginate. This technique offers advantages such as mild reaction conditions, high selectivity, and sensitivity to bioactive substances [153]. In practical applications, the choice of hydrogel preparation method should be guided by the desired physicochemical properties, the degree of crosslinking, and the intended application environment of the hydrogel.

### 4.3. Functionalization of Hydrogel

Variations in the raw materials used to synthesize hydrogels result in a wide range of physicochemical properties, making it difficult for a single type of hydrogel to satisfy the diverse requirements of multiple application scenarios. Consequently, researchers frequently undertake functional modifications to expand the utility of hydrogels. Common strategies for hydrogel functionalization include covalent crosslinking, chemical modifications, immobilization of bioactive molecules, biomimetic modifications, and thermoresponsive alterations [147,156,157,158,159,160]. Through these diverse modification techniques, hydrogels can be endowed with enhanced functionalities, paving the way for versatile and intelligent applications. For instance, covalently crosslinking bioactive monomers with acrylamide monomers can significantly enhance the biocompatibility of acrylamide-based hydrogels [161]. Additionally, by immobilizing specific protein molecules, such as antibodies, onto the hydrogel surface, targeted interactions with particular tissues can be achieved [162]. Cristina Manferdini et al. have reviewed and summarized various strategies for functionalizing hydrogels tailored specifically for OA applications (refer to Table 7) [163].

### 4.4. Strategies Based on Combination of MSCs with Hydrogels for OA Treatment

**Figure 4:**
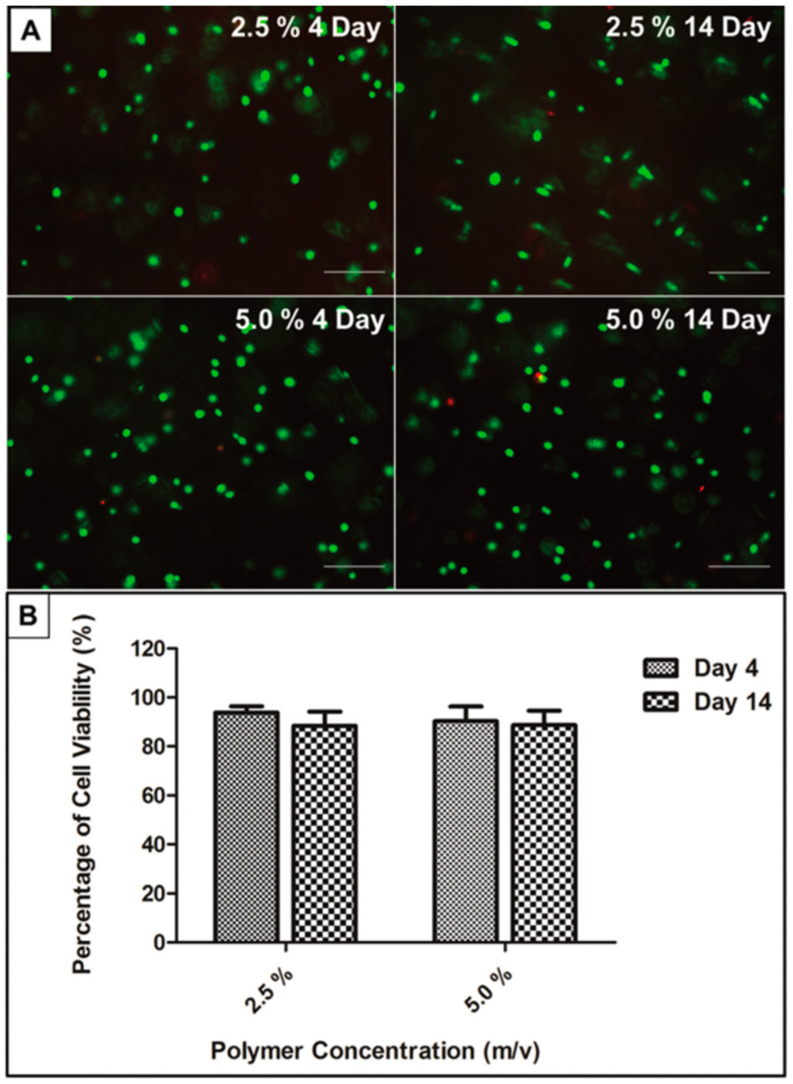
Viability analysis of rASCs in the hydrogels up to 14 days. (**A**) Green staining indicates live cells and red indicates dead cells. (Scale bars represent 100 μm). (**B**) Statistics of (**A**). Adapted with permission from Ref. [174]. Copyright 2010 Royal Society of Chemistry.

**Figure 5:**
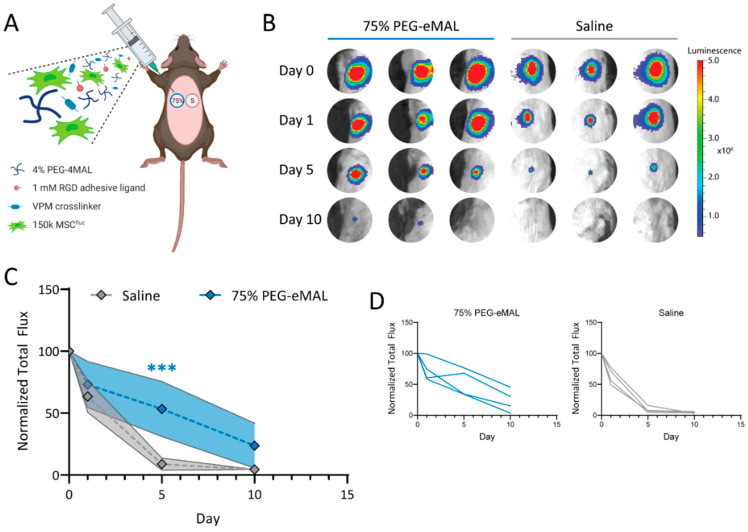
Hydrolytic hydrogels promote MSC retention at site of injection. (**A**) Schematic of MSC retention studies. (**B**) IVIS images of MSC^fluc^ after injection. (**C**) Normalizing the bioluminescence of transplanted MSC^fluc^ over time. *** *p* < 0.001. (**D**) MSC^fluc^ retention over time. (The different line represents each individual sample). Reprinted with permission from Ref. [175]. Copyright 2023 Elsevier.

**Figure 6:**
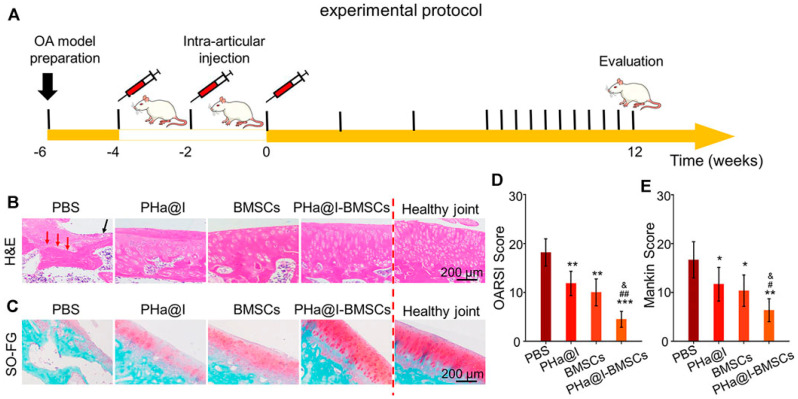
IA injection of BMSCs embedding PHa@I hydrogels prevents cartilage degeneration. (**A**) Schedule of in vivo experiment. (**B**) Hematoxylin eosin staining of cartilage. (**C**) Safranin O-fast green staining of cartilage. (**D**) Osteoarthritis Research Society International (OARSI) scores. (**E**) Markin scores. (“*” vs the PBS group, * *p* < 0.05, ** *p* < 0.01, and *** *p* < 0.001; “#” vs the PHa@I Group, # *p* < 0.05 and ## *p* < 0.01; “&” vs the BMSC Group, & *p* < 0.05). Reprinted with permission from Ref. [178]. Copyright © 2022 Zhu, Ye, Cai, Li, Fan and Yang.

**Figure 7:**
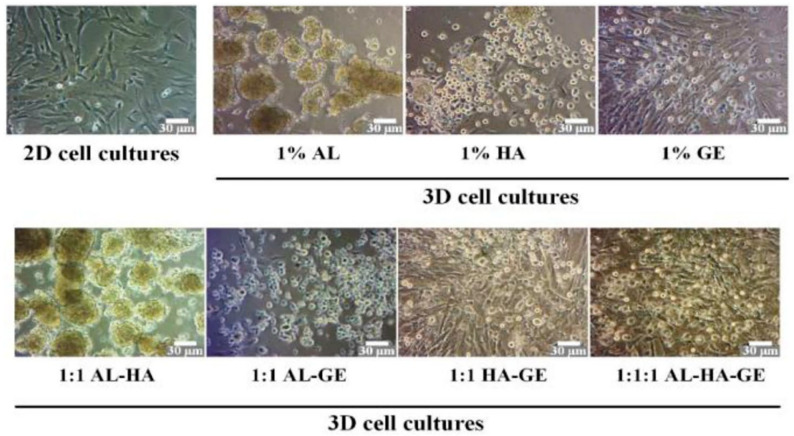
Morphology of MSCs in 3D culture hydrogels. Reprinted with permission from Ref. [195]. Copyright © Amorn Pangjantuk, Palakorn Kaokaen, Phongsakorn Kunhorm, Nipha Chaicharoenaudomrung, and Parinya Noisa 2024.

Hydrogels, with their unique physicochemical properties, offer a promising solution for MSC delivery in OA treatment, effectively addressing several challenges associated with MSCs. Firstly, the internal porous structure of hydrogels provides an optimal environment for cell embedding, ensuring cell survival and functionality. For instance, Dong et al. fabricated a 3D hydrogel system of PEG-based hyperbranched multifunctional homopolymers. They found that that rat AD-MSCs embedded in the hydrogel maintained viability above 85% for two weeks (Figure 4). They also found that when the concentration of the polymer in the hydrogel system is less than 5%, variations in the polymer concentration have no effect on the cell viability [174]. Furthermore, within highly porous hydrogels, MSCs form clusters that enhance cell–cell interactions and prevent cells from diffusing away from the injection site. Karen E. Martin et al. developed a hydrogel-based strategy for MSC delivery for wound healing based on multi-arm poly (ethylene glycol) (PEG) macromers functionalized with maleimide end groups (such as 4-arm macromers, PEG-4MAL). They functionalized PEG-4MAL with an amide or ester to obtained PEG-4aMAL or PEG-4eMAL and constructed hydrogels with varying contents of PEG-4aMAL and PEG-4eMAL to determine the influence of hydrogel formulation on its property. They found that even on day 15, approximately 25% of the MSCs loaded within the 75% PEG-4eMAL hydrogel still resided at the injection site. However, MSCs loaded within the 0% PEG-4eMAL could not be detected on day 5 (Figure 5) [175]. Secondly, hydrogels enhance the anchorage of MSCs, which depend on a nucleated cellular product. Loading MSCs within hydrogels provides a three-dimensional microenvironment that promotes cell–cell interactions and facilitates ECM deposition [176], enabling rapid MSC adherence to the site of cartilage tissue damage. Additionally, encapsulating MSCs within hydrogels can significantly reduce the shear and stretching forces experienced during injection into the joint cavity. In a study led by Matthew A. Wagner and colleagues, the viability of cells in deionized water (as the control) was compared to that of cells in hydrogel during syringe needle flow, aiming to investigate whether hydrogels could effectively prevent cell membrane rupture and subsequently enhance cell viability. They found the viability of cells in the control group decreased by 76.14% from preinjection to postinjection, whereas the viability of cells encapsulated in hydrogel with a high concentration remained stable. Even in hydrogel with a low concentration, cell viability decreased by 19.38% from preinjection to postinjection, which was significantly lower than that observed in the control group [177]. Furthermore, hydrogels can be co-loaded with specific growth factors that promote cell proliferation or differentiation, as well as nutrients that sustain cellular metabolism. Paula Gonzalez-Fernandez et al. covalently conjugated glucose molecules to hyaluronic acid (HA) and used this HA–glucose as a scaffold for constructing an MSC-loaded hydrogel. The presence of ß-glucosidase in the joint cavity allows for the hydrolysis of glycosidic bonds between glucose moieties and hyaluronic acid, resulting in the release of glucose molecules to provide energy for MSCs. In their simulated in vitro experiments, they found that adding glucose to the hydrogel increased MSC viability by 71% [6]. Zhu et al. fabricated a hydrogel delivery system by crosslinking icariin, a component of traditional Chinese medicine known to promote ECM synthesis and enhance the differentiation of MSCs into chondrocytes, with HA. They discovered that the hydrogel incorporating icariin significantly improved the viability of BM-MSCs and induced their differentiation into chondrocytes. Using this carrier system to deliver BM-MSCs in an OA rat model promoted chondrogenesis, inhibited cartilage tissue degradation, and alleviated inflammatory symptoms. Importantly, the therapeutic effect of this carrier system in treating OA was superior to the direct injection of BM-MSCs (Figure 6) [178]. These approaches not only address issues such as insufficient cell proliferation or apoptosis caused by starvation within the joint cavity but also allow for the exploration of optimal therapeutic conditions through varying combinations. Besides, integration of MSCs with advanced gene manipulation techniques [179], such as RNA interference (RNAi) or other gene editing approaches [180] into hydrogels, represents another promising avenue for further improving MSC viability [181,182,183], differentiation capacity [184,185], and tissue repair efficacy [186,187]. Hydrogels serve as an effective platform for the localized and sustained delivery of gene expression modulators, thereby reducing the incidence of off-target effects and ensuring the prolonged regulation of gene expression at the intended target. This approach allows for precise spatiotemporal control over gene expression, enhancing the therapeutic efficacy and safety profile of gene-based interventions [188]. Numerous genes have been identified as pivotal for MSCs’ survival, trophic functions, differentiation potential, immunogenicity, and anti-inflammatory properties (refer to Table 8). For example, research by Shuo Wang and colleagues has highlighted the significance of *Sirtuin 3 (Sirt3)* in sustaining MSC viability under conditions of nutrient deprivation. They discovered that the downregulation of *Sirt3* makes MSCs more susceptible to starvation-induced apoptosis [181]. Consequently, the integration of plasmids that overexpress *Sirt3* into hydrogel scaffolds with MSCs, in conjunction with the controlled release mechanism of these plasmids by the scaffold, has the potential to induce a sustained and elevated expression level of *Sirt3* in MSCs. This approach may confer upon MSCs a heightened resistance to apoptosis triggered by the nutrient-deprived environment within the joint space, thereby enhancing their viability and promoting cartilage tissue regeneration. Similarly, the targeted manipulation of specific gene expression might also improve the adherence of mesenchymal stem cells (MSCs) to the extracellular matrix (ECM) of cartilage [189], reduce the immunogenicity of MSCs [190,191], and promote their chondrogenic differentiation [192]. Indeed, achieving high transfection efficiency in a three-dimensional (3D) system such as a hydrogel may be more straightforward than in a two-dimensional (2D) system. Adriana M. Ledo and colleagues developed a 3D hydrogel system by integrating mesenchymal stem cells (MSCs) with nanocomplexes that contain plasmids encoding for *SOX9* into the hydrogel scaffold. This 3D hydrogel system demonstrated significantly enhanced transfection efficiency for *SOX9* compared to 2D systems. Moreover, the MSCs within this 3D hydrogel exhibited elevated expression levels of chondrogenic markers [193]. Lastly, hydrogels with porous polymeric networks have been demonstrated to mimic stem cell culture environments and promote cell–cell or cell–ECM interactions, providing an ideal medium for MSC expansion. A previous study showed that AD-MSCs cultured within a three-dimensional (3D) hydrogel scaffold enhanced the retention of a rejuvenated population of ASCs that were not senescent, as evidenced by increased expression of “stem-like” surface markers on MSCs compared to two-dimensional (2D) culture systems [194]. Amorn Pangjantuk and colleagues fabricated an alginate–hyaluronic acid (AL-HA) 3D hydrogel culture system. Compared to the 2D monolayer culture, this 3D culture system not only promoted the proliferation and survival of MSCs but also maintained their stemness more effectively. Importantly, MSCs grown within hydrogels exhibit a spherical cellular morphology (Figure 7) [195].

### 4.5. Recent Advances in Application of Combining MSCs with Hydrogel in OA Treatment

The versatility of MSCs has been extensively demonstrated in improving inflammation, preventing chondrocyte apoptosis, and promoting cartilage regeneration in the treatment of OA. In this section, we focus on recent advances in OA treatment that leverage both hydrogels and MSCs.

Currently, AD-MSCs and BM-MSCs are most commonly used in preclinical studies or clinical trials for OA therapy. Nevertheless, due to the limited availability of donor cells, researchers are exploring other potential sources of MSCs. It has been shown that the synovium and synovial fluid within joints contain MSCs [204,205]. MSCs derived from joint tissues are considered to have superior chondrogenic ability. For instance, Jun Li et al. developed a hyper-branched polyPEGDA/HA hydrogel incorporating arthroscopic fluid-derived MSCs (AFF-MSCs). They found that AFF-MSCs possess typical characteristics and properties of MSCs. The viability and DNA content of AFF-MSCs encapsulated in hydrogel respectively doubled and quadrupled over the course of 7 days. Encapsulation of AFF-MSCs promoted expression of chondrogenic markers, which was approximately more than four times that of MSCs cultured in dish. Encapsulated MSCs also exhibited a quadruple efficiency in repairing cartilage defects in rats compared to the control [206]. Additionally, mesenchymal stem cells derived from human umbilical cord blood [207,208] and synovium [209] have also been demonstrated to have therapeutic effects for OA when encapsulated in hydrogels.

**Figure 8:**
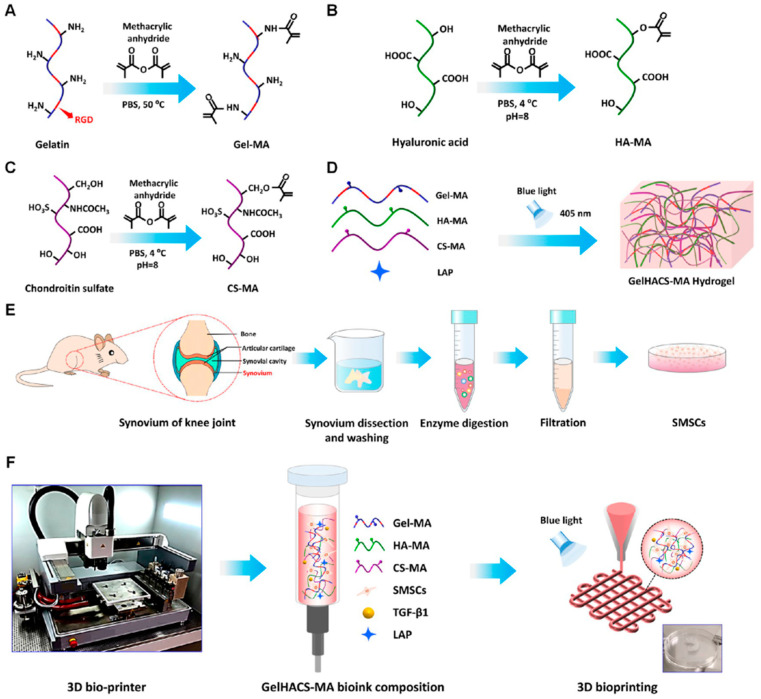
Schematic illustration of the GelHACS-MA hydrogel design. (**A**) Fabrication of Gel-MA. (**B**) Fabrication of HA-MA. (**C**) Fabrication of CS-MA. (**D**) Fabrication of GelHACS-MA hydrogels. (**E**) Isolation of SMSCs. (**F**) Design strategy illustration. Adapted with permission from the Ref. [211]. Copyright 2023 American Chemical Society.

In addition to expanding the sources of MSCs, there is growing interest in using MSCs derived from joint tissues and incorporating extracellular matrix (ECM) components and chondrogenic factors during hydrogel scaffold fabrication. It is believed that MSCs from joint tissues have greater potential for chondrogenic differentiation compared to those from other sources. The inclusion of ECM components endows the hydrogel with properties that mimic the natural ECM, facilitating MSC attachment and growth [210]. The incorporation of chondrogenic factors promotes the differentiation of MSCs into chondrocytes [170]. These elements within the hydrogel work synergistically to promote cartilage regeneration. In a study led by Shengbo Sang, a 3D hydrogel scaffold for regenerating cartilage was constructed by incorporating gelatin methacrylate (Gel-MA), chondroitin sulfate methacrylate (CS-MA), hyaluronic acid methacrylate (HA-MA), transforming growth factor-beta 1 (TGF-β1), and synovium-derived MSCs (SMSCs) (Figure 8). In this system, CS-MA and HA-MA are derivatives of chondroitin sulfate and hyaluronic acid, respectively, which are components of ECM. The incorporation of CS-MA and HA-MA (GelHACS-MA) significantly enhanced the proliferation of MSCs and facilitated cartilage repair in rats. Furthermore, the addition of TGF-β1 (GelHACS-MA + TGF-β1) further augmented the chondrogenic effect of GelHACS-MA [211].

**Figure 9:**
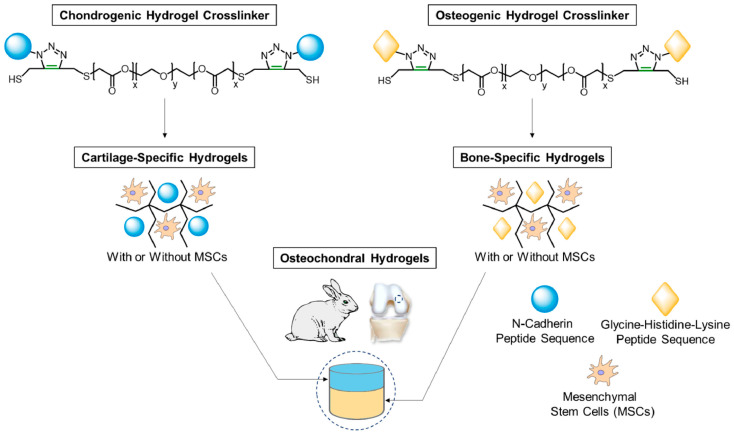
Schematic illustration of regenerating the osteochondral tissue by a bilayered, tissue-specific hydrogel system. Reprinted with permission from Ref. [212]. Copyright 2021 Elsevier.

Advanced OA is characterized not only by damaged cartilage tissue but also by abnormal subchondral bone structure, indicating the presence of osteochondral defects [28]. Therefore, simultaneous recovery of damaged cartilage and subchondral bone tissue is crucial for advanced OA therapy. Jason L. Guo and colleagues developed a bilayered hydrogel system consisting of two distinct layers with a height of 1.5 mm each. The upper layer was bio-conjugated with a chondrogenic peptide (GGGHAVDI) to induce chondrogenesis, while the lower layer was bio-conjugated with a glycine–histidine–lysine peptide derived from osteonectin to induce bone mineralization. Encapsulating MSCs into this bilayered hydrogel system effectively filled up the osteochondral defects in a rabbit model by regenerating both cartilage and bone tissues (Figure 9) [212]. 

## 5. Conclusions and Future Perspectives

Osteoarthritis (OA), a chronic degenerative condition, poses a significant health challenge for hundreds of millions of middle-aged and elderly individuals globally, with a particular impact on women [213]. Existing treatment options such as physical therapy, pharmacotherapy, and surgical intervention offer symptomatic relief but fail to address the underlying cause of the disease and can come with side effects and potential risks [27]. Consequently, researchers are pursuing innovative therapeutic strategies. Among these strategies, mesenchymal stem cell (MSC) therapy has shown considerable promise due to its self-renewal capacity, potential for multilineage differentiation, and low immunogenicity [94]. However, issues such as the low survival rates of MSCs within the joint cavity, their vulnerability to mechanical damage, and loss of stemness during in vitro expansion diminish the therapeutic effectiveness of MSCs and limit their biomedical applications [126,127,128,129].

The use of biodegradable and biocompatible hydrogels has emerged as an innovative strategy for delivering MSCs to the joints to enhance their viability and functionality [22]. These hydrogels simulate the three-dimensional structure of the extracellular matrix, providing an optimal environment for cell growth and protecting cells from mechanical injury [214]. However, there are still some concerns and limitations regarding the application of MSCs. For example, the low immunogenicity of MSCs is considered a crucial factor that cannot be overlooked in their biomedical use. Due to the large quantity required for tissue regeneration, most patients receive allogeneic MSCs during transplantation [215,216]. However, some recent studies found that allogeneic MSCs can elicit immune responses and therefore lead to rejection. Therefore, in addition to starvation and malnutrition, immunological rejection may also be a significant factor contributing to the low viability of MSCs in the articular cavity. Consequently, further investigations are warranted to explore the incorporation of immunosuppressive agents with MSCs into hydrogels for achieving high viability and retention of MSCs in OA treatment [217]. 

Besides, many studies use the term “mimic” to describe the hydrogels’ ability to provide an environment similar to the ECM of cartilage tissues for MSC survival and differentiation. However, in reality, human cartilage tissue is quite rigid and its stiffness exceeds that of commonly used hydrogels in preclinical studies [218]. Additionally, cartilage has a complex architecture, with distinct morphological and structural characteristics, even within the uncalcified cartilage zone [219]. The uncalcified cartilage zone can be divided into three zones based on their structural and constituent variance. For instance, in zone 1, collagen type II fibrils are densely packed, with a thin diameter of about 30–35 nm, and oriented parallel to the surface of the cartilage. In zone 2, the collagen fibrils are thin and oriented obliquely or perpendicularly to the articular surface. Zone 3 has the thickest collagen fibrils with a diameter of 40–80 nm and perpendicular orientation in uncalcified cartilage [220]. Until now, no studies have authentically fabricated hydrogels that structurally and constitutively resemble ECM. Therefore, most hydrogels differ significantly from the ECM of cartilage tissues despite incorporating components such as collagen and proteoglycan. Researchers should exercise caution when using terms like “mimic the ECM of cartilage.” One potential approach to fabricating hydrogels that mimic ECM is by applying 3D printing technology to print different layers similar to actual cartilage. 

Lastly, although many kinds of MSCs derived from different tissues, such as adipose tissues, bone marrow, synovial fluid, and synovial tissues in the joint cavity, have been demonstrated as therapeutic for OA, there is still a lack of studies systematically comparing the accessibility, renewal capacity, chondrogenic differentiation potential, heterogeneity, and immunogenicity among these different types of MSCs. The most suitable MSCs for OA treatment should be identified before combining hydrogels and MSCs on a large scale in future treatments. 

Although there are still challenges in applying hydrogel technology to enhance the therapeutic effect of MSCs in OA treatment, we believe, through these interdisciplinary research initiatives, that safer, more effective, and cost-efficient treatments for OA can be developed, thereby significantly improving the quality of life for patients.

## Figures and Tables

**Table 2:** Identification methods for MSCs.

International Society for Cellular Therapy (ISCT) Definition of MSC Properties	
Plastic adhesion or not	Yes (in standard culture conditions)
Specific antigen	CD105+ CD73+ CD90+ Stro-1+, CD29+, CD44+, CD73+, CD146+, and SSEA4+, CD14-, CD34-, CD45- or CD79a-, CD11b- or HLA-DR-, CD19-
In vitro diffenentiated ability	Adipocytes, osteoblasts, chondroblasts

**Table 4:** Different kinds of MSCs for OA therapy.

Property	Kinds of MSCs	Model	Outcome of OA Treatment	Ref.
Anti- inflammation	IPFP-ASCs	*Human*	Promoting chondrogenic differentiation and preventing articular cartilage thickening and inflammation	[102]
	Supra-hASC	*Mouse*	Reducing OA-associated knee inflammation and cartilage degenerative grade	[103]
	BM-MSCs	*Human*	Promoting cell proliferation of chondrocytes and inhibiting inflammatory activity in osteoarthritis	[104]
	hUCMSCs-EVs	*Human*	Promoting the polarization of M2-type macrophages, reducing the infammatory of cytokines (IL-10) response	[105]
Cartilage regeneration	IPFP-ASCs	*Sheep*	Promoting the expression of cartilage genes	[106]
	BM-MSCs	*Human*	Inducing chondrogenic differentiation	[107]
	Sc-ASCs	*Bear*	Promoting chondrogenic differentiation	[108]
	BM-MSCs	*Human*	Promoting chondrogenic differentiation by enhancing the expression of cartilage extracellular matrix genes	[109]
	BM-MSCs	*Rat*	Prevents cell apoptosis and inhibits senescence of chondrocytes by reducing the IL-1β level and improving the inflammation in joints	[110]

**Table 5:** Types of hydrogels and characterizations.

Types of Hydrogels		Characterizations	Ref.
Natural hydrogels	①Polysaccharide: hyaluronic acid, chondroitin sulfate, chitin, chitosan, cellulose, starch, gum, alginate, and carrageenan	1. Low immune response 2. Low toxicity response 3. Non-toxic and non-immunogenic degradation products 4. Poor stability, rapid degradation 5. Relatively low mechanical strength	[130,133,134,135]
	②Protein-based materials: gelatin, collagen, fibroin, sericin		
	③Polyphenols: lignin		
	④Organic polyester/inorganic polyester: polyphthalamide		
	⑤Polyanhydride: polyadipic acid		
	⑥Biopolymer: nucleic acid, DNA		
Synthetic hydrogels	①Polycaprolactone-(PCL) ②Polyvinylpyrrolidone-(PVP) ③Polylactic acid- (PLA) ④Polyethylene glycol-(PEG) ⑤Polyvinyl alcohol- (PVA)	1. Providing customized performance characteristics 2. Controlability, reproducibility, and excellent mechanical performance 3. Poor compatibility with host tissues 4. Low biological activity	[136,137,138,139,140,141,142]
Hybrid-origin hydrogels	①Carboxymethyl chitosan-(CHC) ②Hyaluronic acid-(HA)	1. pH-dependent drug release characteristics 2. Inhibition of cell apoptosis	[143,144,145]
	①Chitosan ②Polycaprolactone microspheres	1. Dual functionality of supplementing mucus and storing drugs 2. Prolonging drug residence time in the body	
	①Semi-polyacrylonitrile chitosan-poly(acrylamide-ethylene oxide) hydrogel microspheres	Used for encapsulation and delivery of anticancer drugs	

**Table 6:** Hydrogel preparation methods.

Hydrogel Preparation Method	Advantage	Disadvantage	Ref.
Chemical crosslinking	①High degree of crosslinking and stability ②Highly adjustable ③Wide range of applicability	①Biotoxicity ②Toxic substances need to be cleared. ③Long reaction time and complex preparation	[132]
Physical crosslinking	①Mildly reactive and environmentally friendly ②Prepared at room temperature ③Gel structures with reversible properties can be prepared	①Poor gel stabilization ②Sensitive to temperature and ionic concentration conditions and structural instability ③The preparation process can be complex.	[132]
Enzymatic crosslinking	①Good biocompatibility ②Mild chemical reaction, sensitive to biologically active substances ③It can be prepared under physiological conditions.	①Enzyme stability and activity are easily affected. ②The enzyme-catalyzed reaction rate is slower and the preparation time is longer. ③The range of applicability is limited by the available enzymes and substrates.	[154]
Photopolymerization crosslinking	①The preparation process is simple and easy to operate. ②A high degree of crosslinking can be achieved in a relatively short period of time. ③Better spatial and temporal control	①Possible phototoxicity to organisms ②Limited by the depth of light penetration and the rate of reaction ③Technical and equipment support is required for photosensitive monomer selection and light source control.	[155]

**Table 7:** The mechanisms of hydrogels in the treatment of osteoarthritis (OA). Reprinted with permission Ref. [163]. Copyright 2022 MDPI.

Hydrogel Type	Cell Type and Loading	Chondrogenic Inducting Factors	Main Results	Ref.
Fibrin/hyaluronan hydrogel	*Human* BMSCs	TGF-β1	Increasing COL2, ACAN, and GAG levels	[164]
10% PEGDA	*Goat* BMSCs	TGF-β1	Increasing COL2 and GAG level	[165]
Fibrin MeHA	*Human* MSCs	N.I.	Increasing SOX9 level	[166]
DNA supramolecular	*Rabbit* BMSCs	N.I	Increasing COL2, SOX9 and ACAN level, decreasing COL1 and COL10 levels	[167]
PEG–hyaluronic acid (HA)	*Canine* MSCs	TGF-β3	Increaing proteoglycan and GAG levels	[168]
Collagen type 1	*Human* BMSCs	No	Increasing COL2 and GAG levels and condroitin sulfate	[169]
Chondroitin sulfate (CS)	*Rabbit* BMSCs	TGF-β3	Increasing GAG and COL2 levels	[170]
Collagen and alginate	*Human* MSCs	No	Increasing CBFA-1, Sox9, and aggrecan levels	[171]
Chondroitin sulfate (CS) and PEG	*Human* MSCs	N.I.	Increasing collagen II gene expression	[172]
Chitosan	*Rat* BMMSCs	N.I.	Promoting chondrogenesis markers expression (Sox9, aggrecan, and collagen II)	[173]

**Table 8:** Genes that are pivotal for MSCs’ properties.

Gene Symbol	Kinds of MSCs	Gene Functions	Gene Manipulation	Diseases	Ref.
*Sirt3 (sirtuin 3)*	BMSCs	Against starvation-induced apoptosis	Knockdown	In vitro	[181]
*ALKBH5 (AlkB homolog 5)*	BMSCs	Inducer of aging in MSCs	Knockdown	Myocardial infarction	[196]
*circSERPINE2 (serpin family E member 2)*	BMSCs	Inducer of aging in MSCs	Knockdown	Osteoarthritis	[197]
*NICD1 (notch receptor 1)*	BMSCs	Enhanced neuropoietic effects	Knockdown	Ischemic stroke and Parkinson’s	[187]
*ALCAM (activated leukocyte cell adhesion molecule)*	BMSCs	Inhibiting the activation and proliferation of allogeneic CD4^+^ T cells	Knockdown	Allograft rejection, autoimmune diseases	[191]
*SHH (Sonic hedgehog signaling molecule)*	OA-MSC	Inducer of aging in MSCs	Knockdown	Osteoarthritis	[185]
*FOXO1 (forkhead box O1)*	BMSCs	Against TNF-α-induced apoptosis in MSCs	Knockdown	Diabetes	[183]
*TSG-6 (tumor necrosis factor-α-stimulated protein 6)*	HUC-MSCs	Against cellular damage caused by high sugar and fat	Knockdown	Diabetes	[198]
*TREM-2 (triggering receptor expressed on myeloid cells 2)*	MSCs	Critical for MSCs’ pluripotency and immunomodulatory capacity	Knockdown	In vitro	[199]
*TLR4 (Toll-like receptor 4)*	BMSCs	Promote proliferation and osteogenic differentiation of MSCs	Knockdown	Fracture healing, osteoporosis	[184]
*VTN (vitronectin)*	WJ-MSCs	Against starvation-induced apoptosis	Knockdown	Ischemic diseases and wound healing	[182]
*RPS6KA2 (ribosomal protein S6 kinase A2)*	BMSC and UC-MSC	Critical for repairing cartilage defects	Knockdown	Osteoarthritis	[186]
*PUM1 (Pumilio RNA binding family member 1)*	BMSCs	Against aging of MSC	Knockdown	Osteoarthritis	[200]
*LYPLAL1-AS1 (LYPLAL1 antisense RNA 1)*	hADSCs	Against aging of MSC	Overexpression	Senile disease	[201]
*LAMA2 (laminin subunit alpha 2)*	hASCs and hBMMSCs	Inhibiting of osteogenic differentiation but promoting adipogenic differentiation of MSCs	Knockdown	Bone defect diseases	[202]
*HGF (hepatocyte growth factor)*	BMSCs	Repair lung endothelial cell function	Knockdown	Acute lung injury	[203]
*CD44 (CD44 molecule (IN blood group))*	BMSCs	Mediates cell adhesion to ECM, promotes cell migration	Knockdown	Tissue damage and graft fibrosis	[189]
